# Adaptive Emotion Regulation as a Moderator of the Association Between Maladaptive Emotion Regulation and Resilience

**DOI:** 10.7759/cureus.115527

**Published:** 2026-08-31

**Authors:** Pallavi K Kenkare, Changiz Mohiyeddini

**Affiliations:** 1 Psychiatry and Behavioral Sciences, Oakland University William Beaumont School of Medicine, Rochester, USA; 2 Psychology, Oakland University William Beaumont School of Medicine, Rochester, USA

**Keywords:** adaptive emotional regulation, emotion regulation, maladaptive emotional regulation, psychological resilience, regulatory repertoire

## Abstract

Introduction

Emotional regulation strategies operate not in isolation, but within a broader regulatory repertoire. Under this lens, this paper investigates whether adaptive cognitive emotion regulation strategies moderate the relationship between maladaptive strategies and psychological resilience.

Methods

In a sample of 69 adults (mean age = 26.2 years; 75.36% female), adaptive and maladaptive cognitive emotion regulation were assessed using the Cognitive Emotion Regulation Questionnaire (CERQ), and resilience was measured using the Brief Resilience Scale (BRS).

Results

Moderation analysis using Hayes’s PROCESS macro (Model 1) revealed a significant interaction between maladaptive and adaptive emotion regulation in predicting resilience (ΔR² = 0.085, p = 0.012). Specifically, greater maladaptive emotion regulation was significantly associated with lower resilience at low levels of adaptive emotion regulation (b = -0.499, p = 0.009), whereas the association was nonsignificant at median (b = -0.221, p = 0.157) and high (b = -0.082, p = 0.622) levels of adaptive emotion regulation.

Conclusion

These findings suggest that adaptive emotion regulation moderates the association between maladaptive emotion regulation and resilience, with higher levels of adaptive regulation associated with a weaker negative relationship. The findings are consistent with a buffering moderation pattern and support further longitudinal and experimental investigation of adaptive regulation capacity as a potential target in resilience-focused interventions.

## Introduction

Psychological resilience - the capacity to maintain or rapidly recover adaptive functioning in the face of adversity - is shaped by how individuals cognitively process and regulate their emotional responses to stressful events [1,2]. Cognitive emotion regulation is the conscious cognitive management of processing emotionally stimulating information [3]. Adaptive strategies, such as positive reappraisal (assigning positive meaning or growth to an event), acceptance (accepting the occurance of the event), putting into perspective (contextualizing the event amongst other experiences), positive refocusing (shifting attention towards positive thoughts), and refocus on planning (thinking about next steps to manage the event), have been consistently linked to positive psychological outcomes including resilience, wellbeing, and reduced psychopathology [4,5]. On the other hand, maladaptive strategies, such as rumination (focusing upon thoughts and feelings related to the event), catastrophizing (over-emphasizing negative aspects of the event), other-blame (attributing responsibility for the event to others), and self-blame (attributing responsibility for the event to oneself), have been associated with anxiety, depression, and impaired coping [3,4].

The regulatory repertoire lens proposes that the use of any single emotion regulation strategy exists not in isolation, but in the context of an individual’s broader regulatory repertoire [6]. Thus, some studies have postulated that the harm associated with maladaptive strategies may be attenuated when an individual simultaneously possesses and deploys a meaningful repertoire of adaptive alternatives [7]. Essentially, when adaptive strategies are developed and regularly utilized, they may serve as a corrective counterforce, constructively re-orienting cognitive processing, even in the presence of maladaptive tendencies. Conversely, when adaptive strategies are absent or underutilized, maladaptive processing operates without check.

The regulatory repertoire lens informs this paper’s hypothesis: Adaptive cognitive emotional regulation strategies will moderate the relationship between maladaptive strategies and psychological resilience, such that the negative association between maladaptive strategies and resilience will be stronger at lower levels of adaptive regulation and attenuated as adaptive regulation rises.

To our knowledge, the specific interaction between these two distributions of cognitive emotional regulation upon predicting resilience has not been directly tested. The independent contributions and correlations of adaptive and maladaptive emotion regulation strategies upon resilience have been well examined [8,9]. However, little attention has been paid to how these two regulatory poles interact with one another. Adaptive and maladaptive strategies have been examined as independent predictors, as correlated dimensions, or as competing variables in regression models, but their interaction as a moderation effect on resilience outcomes has not been examined. This paper addresses the gap by evaluating whether adaptive emotional regulation strategies moderate the relationship between maladaptive strategies and psychological resilience.

## Materials and methods

Participants and recruitment

This cross-sectional survey, open from March 2025 to March 2026, recruited participants using non-probability convenience sampling through social media platforms (Facebook, LinkedIn, Reddit) and a North American university psychology research pool. The study was conducted online as a survey via the Qualtrics platform (Qualtrics, Provo, Utah, USA). Our sample comprised 69 adults (mean age = 26.2, standard deviation (SD) = 6.0; range 21-59 years; 75.36% female, 21.74% male, 2.90% non-binary or third gender). A formal a priori power analysis was not conducted. A recruitment target of approximately 100 participants was established prospectively based on investigator judgment and study feasibility. The final sample reflected eligible participants who completed the study during the predetermined recruitment period. Participants were eligible if they were ≥18 years old, able to read English, and provided informed consent electronically at the beginning of the survey. Participants with incomplete data on the primary study measures required for the moderation analysis were excluded from the analytic sample. Only participants with complete data on the primary study measures required for the moderation analysis were included in the final analytic sample (N = 69). This study was determined to be exempt by the Oakland University Institutional Review Board (IRB-FY2025-234).

Measures

Psychological Resilience

The Brief Resilience Scale (BRS) assessed the ability to recover from stress across six items rated on a 5-point Likert scale (1 = strongly disagree, 5 = strongly agree) [2]. Higher scores indicate greater resilience (mean = 3.42, SD = 0.95; α = 0.92). After reverse-scoring the negatively worded items, item responses were averaged so that higher scores indicated greater resilience. Scores therefore ranged from 1 to 5.

Cognitive Emotion Regulation

The Cognitive Emotion Regulation Questionnaire (CERQ), a self-report survey, identified and measured participants' use of regulation strategies, as well as differentiated said strategies into adaptive and maladaptive strategies [3]. The CERQ assessed cognitive regulation strategies using nine subscales [3]. The Adaptive Emotion Regulation composite (CERQAd) evaluated positive reappraisal, acceptance, putting into perspective, positive refocusing, and refocus on planning subscales [3]. The Maladaptive Emotion Regulation composite (CERQMal) evaluated rumination, catastrophizing, other-blame, and self-blame subscales [3]. Items were rated on a 5-point Likert scale and averaged to produce composite scores ranging from 1 to 5. For the present analysis, these subscales were grouped into adaptive and maladaptive composite scores in accordance with their classification in the emotion-regulation literature and the regulatory repertoire framework. These composite scores were used as analytic groupings for the present study rather than treated as independently validated higher-order CERQ factors. In the present sample, adaptive emotion regulation had a mean of 3.46 (SD = 0.71; α = 0.85), and maladaptive emotion regulation had a mean of 2.79 (SD = 0.75; α = 0.84).

Analytic strategy

Descriptive statistics, including means, SDs, and ranges, were calculated for the primary study variables and CERQ subscales. Internal consistency of the BRS and adaptive and maladaptive CERQ composites was assessed using Cronbach’s alpha. Pearson product-moment correlations were used to assess bivariate associations among resilience, adaptive emotion regulation, and maladaptive emotion regulation.

Prior to regression analysis, assumptions of linearity, homoscedasticity, normality of residuals, and multicollinearity were evaluated. Linearity and homoscedasticity were assessed by visual inspection of standardized residuals plotted against standardized predicted values, with homoscedasticity additionally evaluated using the Breusch-Pagan test. Normality of residuals was assessed using the Shapiro-Wilk test, residual plots, skewness, and kurtosis. Multicollinearity was assessed using tolerance values and variance inflation factors (VIFs).

Moderation analysis was conducted using Hayes’s PROCESS macro for IBM SPSS Statistics for Windows, Version 32 (Released 2026; IBM Corp., Armonk, New York, United States) [10]. Resilience was entered as the dependent variable, maladaptive emotion regulation as the focal predictor, and adaptive emotion regulation as the moderator. Continuous variables constituting the interaction term were mean-centered prior to analysis to facilitate interpretation of lower-order effects. The interaction term was calculated as the product of the centered maladaptive and adaptive emotion-regulation scores. HC3 heteroscedasticity-consistent standard errors were used to provide robust inference. The contribution of the interaction term was assessed using the change in explained variance (ΔR²). Conditional effects of maladaptive emotion regulation on resilience were probed at the 16th, 50th, and 84th percentiles of adaptive emotion regulation [11]. All statistical tests were two-tailed, with α = 0.05.

## Results

Descriptive statistics and preliminary analyses

Out of the 69 individuals who finished the survey, 66 reported their age, with an average age of 26.2 and a range from 21 to 59 years old. Educational achievement was high, with 81.2% of individuals achieving a Bachelor's degree, and 17.4% of individuals earning a graduate or professional degree. Survey participants were predominantly female, with 75.36% of participants identifying as female. All sociodemographic data are presented in Table 1.

**Table 1:** Sociodemographic Characteristics of Participants (N = 69) * Age data were available for 66 participants; three participants did not provide age information. GED: General Educational Development

Characteristic	n (%) or Mean ± SD
Age, years (N = 66)*	26.2 ± 6.00
Range	21-59
Gender (N = 69)	
Female	75.36%
Male	21.74%
Non-binary/third gender	2.90%
Education (N = 69)	
High school diploma or GED	1.45%
Bachelor’s degree	81.16%
Graduate or professional degree	17.39%

Descriptive statistics for key study variables are presented in Table 2.

**Table 2:** Descriptive Statistics for Key Study Variables (N = 69) BRS: Brief Resilience Scale; CERQAd: Adaptive Cognitive Emotion Regulation Questionnaire composite; CERQMal: Maladaptive CERQ composite

	N	Minimum	Maximum	Mean	Std. Deviation
BRS (Resilience)	69	1	5	3.4203	0.95084
CERQAd (Adaptive Emotional Regulation)	69	1	4.8	3.4551	0.70451
Subscale: Acceptance	69	1	5	3.6667	1.0278
Subscale: Positive Refocusing	69	1	5	2.8986	0.85138
Subscale: Refocus on Planning	69	1	5	3.7174	0.9451
Subscale: Positive Reappraisal	69	1	5	3.4855	1.09131
Subscale: Putting into Perspective	69	1	5	3.5072	1.03075
CERQMal (Maladaptive Emotional Regulation)	69	1	5	2.788	0.75254
Subscale: Self Blame	69	1	5	2.9855	1.13102
Subscale: Rumination	69	1	5	3.2971	1.07894
Subscale: Catastrophizing	69	1	5	2.5435	0.97671
Subscale: Other Blame	69	1	5	2.3261	0.93866

Regression diagnostics indicated no substantial violation of linearity or homoscedasticity based on visual inspection of standardized residuals plotted against standardized predicted values. The Breusch-Pagan test provided no evidence of heteroscedasticity, χ²(3) = 4.01, p = 0.260. Tolerance values ranged from 0.768 to 0.860 and VIFs from 1.16 to 1.30, indicating no problematic multicollinearity. The Shapiro-Wilk test indicated a statistically significant departure from normality of residuals (W = 0.945, p = 0.004); however, residual skewness (-0.43) and kurtosis (-0.84), together with visual inspection of the residual plots, suggested that the departure was modest.

Pearson product-moment correlations indicated that adaptive and maladaptive emotion regulation were positively correlated (r = 0.29, p = 0.015), suggesting that greater use of adaptive strategies may coexist with greater use of maladaptive strategies rather than the two representing opposite ends of a single continuum. Adaptive emotion regulation was positively correlated with resilience (r = 0.38, p = 0.001), whereas maladaptive emotion regulation showed a nonsignificant negative correlation with resilience (r = -0.16, p = 0.199). Findings are presented in Table 3.

**Table 3:** Pearson Correlations among Study Variables * = p < 0.05, and ** = p < 0.01 CERQMal: Maladaptive Cognitive Emotion Regulation; CERQAd: Adaptive Cognitive Emotion Regulation Questionnaire composite

Variable	Statistic	CERQMal	Resilience	CERQAd
CERQMal	Pearson Correlation	1	-0.157	0.292*
	Sig. (2-tailed)	-	0.199	0.015
Resilience	Pearson Correlation	-0.157	1	0.377**
	Sig. (2-tailed)	0.199	-	0.001
CERQAd	Pearson Correlation	0.292*	0.377**	1
	Sig. (2-tailed)	0.015	0.001	-
	N	69	69	69

Moderation analysis

The overall moderation model was statistically significant (R² = 0.305, FHC3​(3, 65) = 10.75, p < 0.001), indicating that the three predictors together accounted for approximately 30.5% of the variance in resilience. At the mean level of adaptive emotion regulation, the association between maladaptive emotion regulation and resilience was negative but not statistically significant (b = -0.271, SE(HC3) ​= 0.155, t(65) = -1.75, p = 0.084, 95% CI (-0.580, 0.038)). Adaptive emotion regulation was positively associated with resilience at the mean level of maladaptive emotion regulation (b = 0.785, SE(HC3) ​= 0.161, t(65) = 4.87, p < 0.001, 95% CI (0.463, 1.107)).

Critically, the interaction between maladaptive and adaptive cognitive emotion regulation was statistically significant (b = 0.348, SE(HC3) ​= 0.134, t(65) = 2.60, p = 0.012, 95% CI (0.080, 0.615)). The interaction accounted for an additional 8.5% of explained variance, ΔR^2^ = 0.085, FHC3​(1,65) = 6.74, p = 0.012, consistent with H1. Full regression results are presented in Table 4.

**Table 4:** Moderation Analysis: Maladaptive Cognitive Emotional Regulation Strategies (CERQMal) × Adaptive Cognitive Emotional Regulation Strategies (CERQAdap) Predicting Resilience (N = 69) Model: R^2^ = 0.305, FHC3​(3,65) = 10.75, p < 0.001; Interaction: ΔR^2^ = 0.085, FHC3​(1,65) = 6.74, p = 0.012. Note: Continuous predictors were mean-centered prior to construction of the interaction term. HC3 heteroscedasticity-consistent standard errors are reported. ΔR^2^ = incremental R-squared for the interaction term. 95% confidence intervals (CIs) are reported. N = 69. LLCI: lower limit of the 95% CI; ULCI: upper limit of the 95% CI

	b	SE(HC3)	t	p	LLCI	ULCI
constant	3.3672	0.1029	32.7107	p < 0.001	3.1617	3.5728
CERQMal	-0.271	0.1545	-1.7537	0.0842	-0.5796	0.0376
CERQAdap	0.7851	0.1611	4.8722	p < 0.001	0.4633	1.1069
Interaction	0.3476	0.1339	2.5962	0.0116	0.0802	0.615

Probing the interaction at the 16th, 50th, and 84th percentiles of adaptive emotion regulation revealed a graded pattern of conditional effects (Table 5 and Figure 1). At low adaptive emotion regulation (16th percentile; raw CERQAd mean = 2.80), greater maladaptive emotion regulation was significantly associated with lower resilience (b = -0.499, SE(HC3)​ = 0.185, t(65) = -2.70, p = 0.009, 95% CI (-0.867, -0.130)). At the median level of adaptive emotion regulation (50th percentile; raw CERQAd mean = 3.60), the negative association was smaller and no longer statistically significant (b = -0.221, SE(HC3)​ = 0.154, t(65) = -1.43, p = 0.157, 95% CI (-0.528, 0.087)). At high adaptive emotion regulation (84th percentile; raw CERQAd mean = 4.00), the association was further attenuated and remained nonsignificant (b = -0.082, SE(HC3)​ = 0.165, t(65) = -0.50, p = 0.622, 95% CI (-0.411, 0.248)). Overall, this pattern was consistent with a buffering moderation effect, such that the negative association between maladaptive emotion regulation and resilience became weaker at higher levels of adaptive emotion regulation.

**Table 5:** Conditional Effects of Maladaptive Cognitive Emotional Regulation Strategies (CERQMal) on Resilience at Values of Adaptive Cognitive Emotional Regulation Strategies (CERQAd) (N = 69) Moderator values correspond to the 16th, 50th, and 84th percentiles of the adaptive emotional regulation distribution. Effects represent the unstandardized regression coefficient for CERQMal on resilience (BRS) at each level of adaptive emotional regulation. LLCI: lower limit of the 95% confidence interval (CI); ULCI: upper limit of the 95% CI; BRS: Brief Resilience Scale

CERQAd Level	CERQAd Value	b	SE(HC3)	t(65)	p	LLCI	ULCI
Low (16th pct)	-0.655	-0.499	0.185	-2.70	0.0087	-0.867	-0.130
Median (50th pct)	+0.145	-0.221	0.154	-1.43	0.1567	-0.528	0.087
High (84th pct)	+0.545	-0.082	0.165	-0.50	0.622	-0.411	0.248

**Figure 1:**
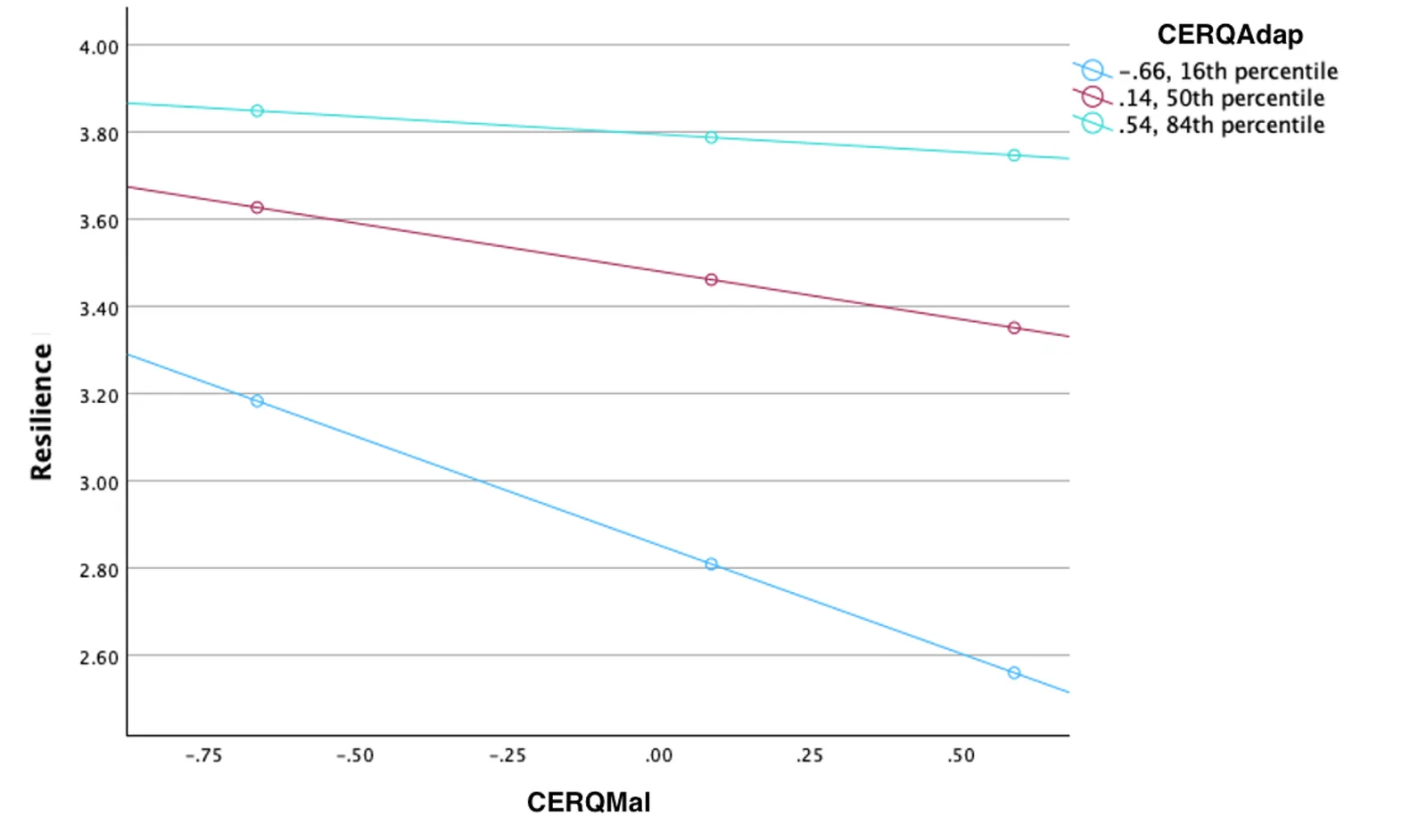
Interaction Plot Describing Moderation Analysis CERQMal: Maladaptive Cognitive Emotional Regulation Strategies; CERQAdap: Adaptive Cognitive Emotional Regulation Strategies

## Discussion

This study tested whether adaptive cognitive emotion regulation moderates the relationship between maladaptive regulation and psychological resilience using a sample of 69 adults. The findings were consistent with a significant buffering interaction: the negative association between maladaptive emotion regulation and resilience was strongest when adaptive emotion regulation was low and progressively attenuated as adaptive emotion regulation increased, becoming non-significant at median and high levels of adaptive emotion regulation. The interaction accounted for an additional 8.5% of variance in resilience and remained statistically significant using HC3 robust standard errors. These findings advance understanding of how adaptive and maladaptive cognitive emotion regulation strategies may interact in relation to resilience outcomes, with implications for future research on therapeutic intervention.

Theoretical implications

The finding that adaptive cognitive emotion regulation moderated the association between maladaptive strategies and resilience provides further support for the regulatory repertoire viewpoint. As previously stated, the regulatory repertoire hypothesis proposes that individual psychological outcomes are impacted not by any sole regulation strategy in isolation, but by interactions between and in the context of an individual’s overall inventory of emotional regulatory mechanisms [6]. These results support and add another facet to this framework; specifically, they suggest that the association between maladaptive emotion regulation and resilience differs depending on the development of the adaptive emotion regulation skills present within an individual’s regulatory repertoire. This interpretation is also consistent with the positive correlation observed between adaptive and maladaptive emotion regulation in the present study. Rather than functioning as opposite ends of a single continuum, adaptive and maladaptive strategies may coexist within an individual’s regulatory repertoire. Accordingly, the use of maladaptive strategies does not necessarily imply an absence of adaptive strategies, and vice versa.

The progressive attenuation of the association between maladaptive emotion regulation and resilience warrants emphasis. At low levels of adaptive emotion regulation, maladaptive emotion regulation was significantly associated with lower resilience (b = -0.499). At median levels of adaptive regulation, this association was reduced in magnitude and no longer statistically significant (b = -0.221), and at high levels it was further attenuated (b = -0.082). Because these coefficients are unstandardized, their numerical magnitude is not interpreted according to conventional small, medium, or large effect-size categories. Rather, the pattern indicates that the negative association between maladaptive emotion regulation and resilience became progressively weaker as adaptive emotion regulation increased.

This suggests that the types of emotion regulation strategies an individual possesses in their repertoire may matter in the context of what else is available within that repertoire. An individual who ruminates but also actively utilizes reappraisal may therefore occupy a different regulatory context than one who ruminates without frequently utilizing adaptive strategies. The present findings consequently support considering emotion regulation as a broader repertoire of co-occurring strategies rather than evaluating maladaptive strategies in isolation.

The present findings are broadly consistent with prior research linking cognitive emotion regulation strategies with resilience and psychological outcomes. Previous studies have found that adaptive cognitive emotion regulation strategies were more strongly associated with resilience than maladaptive strategies in patients with depression and anxiety, with greater use of refocus on planning and positive reappraisal and lower use of rumination associated with greater resilience [8]. Similarly, prior work examining adaptive and maladaptive strategies together has suggested that their relevance to psychological outcomes may depend on the broader pattern of strategies available to the individual [7]. To our knowledge, however, prior studies have not directly tested whether adaptive cognitive emotion regulation moderates the association between maladaptive cognitive emotion regulation and resilience. The present findings extend this literature by demonstrating that the negative association between maladaptive regulation and resilience varied as a function of adaptive regulation, becoming progressively weaker at higher levels of adaptive strategy use.

Practical implications

Our findings may have implications for future resilience-building and emotion regulation interventions, particularly in populations characterized by maladaptive emotion regulation patterns. The present findings suggest that it may be useful for future research to examine and invest in the role of the adaptive emotion regulation skills within the broader regulatory repertoire

Importantly, the present study did not examine the reduction of maladaptive emotion regulation, manipulate adaptive or maladaptive strategies, or evaluate treatment response. Therefore, these findings cannot establish whether reducing maladaptive regulation or increasing adaptive regulation would improve resilience. Rather, the data demonstrate that the association between maladaptive regulation and resilience differed according to the level of adaptive regulation: maladaptive regulation was significantly associated with lower resilience when adaptive regulation was low, whereas this association was not statistically significant at median or high levels of adaptive regulation.

Future intervention research may therefore benefit from examining whether building adaptive emotion regulation skills alters this association over time. This perspective shifts attention from deficit reduction alone towards additional resource building. Rather than asking only, “How do we decrease individual use of harmful strategies?”, future work might also ask, “How do we ensure individuals have strong adaptive alternatives available in times of need?” In time-limited educational, occupational, and clinical settings, this shift could have many meaningful practical implications.

Strengths

This study has several strengths. First, it examined adaptive and maladaptive emotion regulation simultaneously, allowing their interaction to be tested directly rather than treating them as isolated or mutually exclusive variables. This approach is particularly relevant to the regulatory repertoire framework and allows examination of whether the association of one form of regulation depends on the presence of another.

Second, the measures demonstrated good to excellent internal consistency in the present sample, with Cronbach’s alpha values of 0.92 for resilience, 0.85 for adaptive emotion regulation, and 0.84 for maladaptive emotion regulation. Third, the moderation effect was probed at the 16th, 50th, and 84th percentiles of adaptive emotion regulation, allowing the conditional nature of the association to be characterized rather than relying solely on the statistical significance of the interaction term.

Limitations and future directions

Several limitations warrant acknowledgment. First, given the cross-sectional design of our study, we cannot state the causality or temporal directionality among the observed associations. Although the moderation model specified adaptive emotion regulation as moderating the association between maladaptive emotion regulation and resilience, we cannot conclude that adaptive emotion regulation causally protects resilience from the effects of maladaptive regulation. Reverse or bidirectional relationships are also possible [9]. Further longitudinal and experimental designs are needed to evaluate the causal mechanisms underlying these relationships.

Second, the sample was predominantly female (75.36%), had high educational attainment, and was recruited using non-probability convenience sampling that included a university research pool. These factors limit generalizability to broader and more diverse populations.

Third, with N = 69, the analytic sample was relatively modest in size, and the recruitment target was not derived from a formal a priori power analysis. Although the primary moderation interaction was statistically significant, this does not establish that the study was adequately powered to detect the planned interaction, particularly for smaller effects. The modest sample may also reduce the precision and stability of the estimated moderation effect. Replication of this design with larger, prospectively powered samples is warranted.

Finally, regression residuals demonstrated a statistically significant but modest departure from normality. Other regression diagnostics did not indicate substantial violations of linearity, homoscedasticity, or multicollinearity, and the moderation interaction remained statistically significant using HC3 robust standard errors. Nevertheless, the residual departure from normality and relatively modest sample size warrant cautious interpretation and replication of the findings.

Future research should also examine whether the observed moderation pattern can be generalized to specific maladaptive strategies beyond the CERQMal composite, such as self-blame, rumination, catastrophizing, and other-blame examined separately, as well as to individual adaptive strategies within the CERQAd composite. Further studies should also investigate whether similar conditional associations are observed for other resilience-related and clinically relevant outcomes, including anxiety, depression, burnout, and broader indicators of psychological functioning.

## Conclusions

This study found that adaptive cognitive emotion regulation moderated the association between maladaptive emotion regulation and resilience. When adaptive emotion regulation levels were low, greater maladaptive emotion regulation was significantly associated with lower resilience; at median and high levels of adaptive emotion regulation, this negative association was attenuated and no longer statistically significant. These findings are consistent with a buffering moderation pattern in which the association between maladaptive regulation and resilience varies according to the broader adaptive regulatory context.

Our results contribute to the regulatory repertoire model of emotion regulation and resilience, in which outcomes may depend on the interplay among regulatory resources rather than on individual strategies considered in isolation. These findings support further longitudinal and experimental research examining whether strengthening adaptive emotion regulation can influence resilience-related outcomes, and may ultimately help inform therapeutic efforts in educational and clinical settings.
